# Hepatic cerebrospinal fluid pseudocyst mimicking hydatid liver disease: a case report

**DOI:** 10.1186/1752-1947-5-475

**Published:** 2011-09-23

**Authors:** Walid Faraj, Houssein Haidar Ahmad, Deborah Mukherji, Mohamed Khalife

**Affiliations:** 1 HBP and Liver Transplant Unit, Department of Surgery, American University of Beirut-Medical Centre, American University of Beirut Street, Beirut-Lebanon

## Abstract

**Introduction:**

An abdominal pseudocyst is a rare complication of a ventriculo-peritoneal shunt. Etiological factors include infection, obstruction and dislodgement. This is the first report of a hepatic cerebrospinal fluid pseudocyst mimicking hydatid liver disease.

**Case presentation:**

We report the case of an 18-year-old Caucasian male patient who presented with a hepatic pseudocyst secondary to a ventriculo-peritoneal shunt, misdiagnosed as hydatid disease of the liver.

**Conclusion:**

Hepatic pseudocysts, a rare complication of a ventriculo-peritoneal shunt, have similar clinical and radiological characteristics to those of hydatid liver disease. The formation of a pseudocyst should always be considered in patients with ventriculo-peritoneal shunts *in situ*.

## Introduction

An abdominal pseudocyst is a rare complication of a ventriculo-peritoneal shunt. Such cysts may cause diagnostic problems in regions such as the Middle East, where echinococcosis disease of the liver is endemic, due to similarities in clinical presentation and radiological appearance.

## Case presentation

An 18-year-old Caucasian male patient presented with a 10-day history of generalized tonic-clonic seizures. His past medical history included right ventriculo-peritoneal (VP) shunt insertion at two weeks of age for bacterial meningitis complicated by hydrocephalus. Four years prior to his current admission he had presented with abdominal pain and a computed tomography (CT) scan of his abdomen at that time was interpreted as being consistent with a right hepatic hydatid cyst (8 × 6 cm). Serology workup was negative for hydatid disease at the time of the CT scan, however, due to the characteristic radiological findings, antihelminthic treatment (albendazole) was commenced and he was subsequently lost to follow-up.

**Figure 1:**
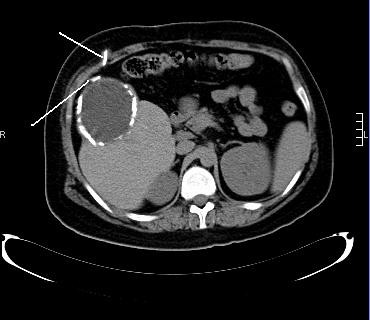
**Abdominal CT scan showing 11 × 9 cm pseudocyst of his right hepatic lobe, with peripheral calcifications and the tip of the VP shunt going inside the cyst (arrow)**.

**Figure 2:**
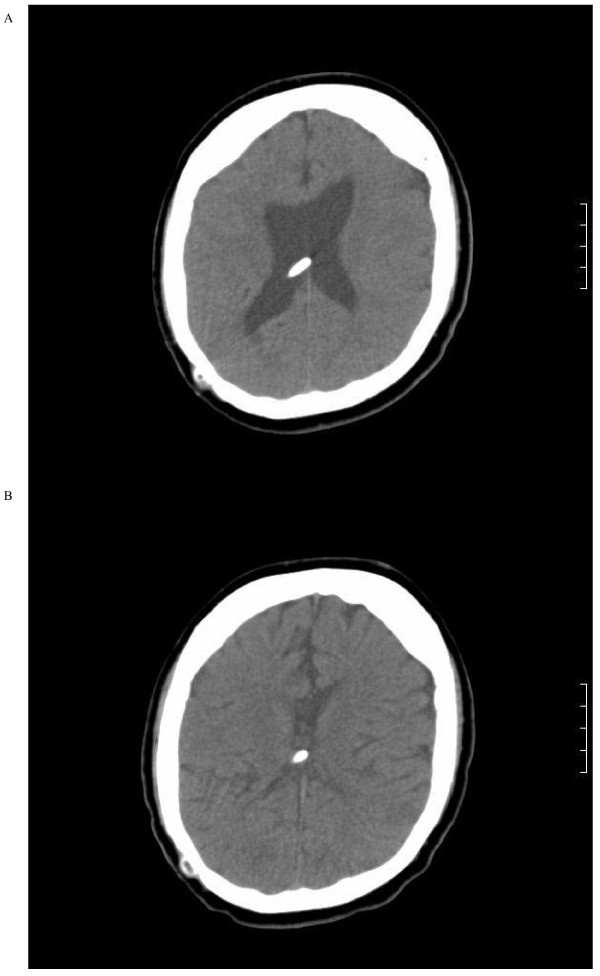
**CT scan of the patient's brain**. (A) Preoperative ventricular dilatation. (B) Postoperative decompression of the ventricle

During his current admission, he underwent a CT scan of his brain and abdomen, which revealed an increase in the size of the cerebral ventricles and an increase in the size of the liver cyst(11 × 9 cm), revealing the presence of the VP shunt tip inside the cyst (Figure 1). Exploratory laparotomy was performed and the tip of the shunt was found inside the cyst, which was opened and drained. The hepatic cyst was found to contain cerebrospinal fluid with no evidence of hydatid disease. The VP shunt was repositioned in his pelvis; our patient made an excellent postoperative recovery and was discharged home after four days. A follow-up CT scan showed regression of the dilated cerebral ventricles (Figure 2a, b).

## Discussion

VP shunts are foreign bodies that may cause intra-abdominal complications. Major intra-abdominal complications include ascites, peritoneal infections, intestinal obstructions and perforations, pseudocyst, abscess formation and inguinal hernia. Migration of the distal catheter and metastases of brain tumor have also been reported [1-3]. The incidence of intra-abdominal cerebrospinal fluid (CSF) pseudocyst varies between 1% and 3% in different studies. Hepatic pseudocyst secondary to a shunt is extremely rare [4,5].

The formation of an abdominal CSF pseudocyst was first described by Harsh in 1954 [6]. Non-specific clinical presentations may cause diagnostic and therapeutic difficulties; physicians should be aware of this complication, especially in unconscious patients [7]. The precise etiology for abdominal pseudocyst formation is still unknown. An inflammatory process, either sterile or infectious, is generally regarded as the main causative factor [8-10]. Other predisposing factors have been postulated such as peritonitis, prior surgical peritoneal adhesion, a history of central nervous system (CNS) infections and CNS tumors, distal shunt migration, multiple shunt revisions, malabsorption of CSF and allergic reaction [10,11].

The time between last VP shunt operation and development of an abdominal pseudocyst has been reported from three weeks to 10 years [12].

Hepatic pseudocysts secondary to VP shunts are classified as intra-axially or extra-axially growing pseudocysts when penetrating the Glisson capsule; the shunt tube can cause extra-axial subcapsular pseudocyst formation [13]. Alternatively, the tip of the shunt can be lodged in the liver parenchyma and cause formation of an intra-axially growing pseudocyst deep within the parenchyma [14]. The most important factors causing hepatic pseudocyst are migration of the peritoneal tip of the shunt to the liver surface and its chronic irritation [7]. Consequently, the oncotic pressure of the cystic fluid increases, interstitial fluid passes into the cyst, and the cyst increases in size [2].

Hydatid disease is endemic in the Middle East. The combination of imaging and serology are usually used to make the diagnosis. In this case, the initial diagnosis was misled by the radiological findings of peripheral calcifications that may represent the common appearance of hydatid cyst.

Imaging findings of echinococcosis reflect a spectrum depending on the developing stages of the parasitic cyst in the human tissue, ranging from a single unilocular cyst, to multiple daughter cyst formation, and then gradually to a solid and calcified cyst [15].

The fact that the hydatid indirect hemagglutinin (IHA) test was negative does not rule out hydatid disease however, in retrospect, would make a peritoneal cyst or pseudocyst as likely. The sensitivity and specificity of the IHA are 86.7% and 95% respectively [16]. Only a positive hydatid serology is valuable; a negative serologic test does not exclude the diagnosis [17].

The suspicion of an abdominal pseudocyst is often made at the time of physical examination and on the basis of conventional radiology [7]. Visualization of the distal tip of the VP shunt within a homogeneous intraperitoneal collection is the principal diagnostic sign of an abdominal CSF pseudocyst on ultrasound and CT. Ultrasonography is the method of choice because it is fast and reliable [8]. However, a CT scan of the abdomen provides a more accurate diagnosis [18]. For the diagnosis of an extra-axially growing hepatic pseudocyst, abdominal CT images are typical. The pseudocyst is surrounded by an annulus showing continuity with hepatic tissue [13].

The standard treatment of a hepatic pseudocyst secondary to catheter tip migration, in cases with no infection or prominent inflammatory reaction in the peritoneal cavity, should be simple repositioning of the peritoneal catheter in the abdominal cavity. This procedure may be combined with percutaneous or open drainage in resistant cases [3]. However, there are numerous therapeutic approaches for the management of shunt-related abdominal pseudocysts reported in the literature [19]; simple aspiration of the cyst under CT or ultrasound guidance [5]; removal of the shunt and installation of a new one once the cyst is resolved; if it is not resolved, shunt revision following cyst aspiration; or draining the cyst fluid through a explorative laparotomy, unroofing the cyst wall, then shunt revision or repositioning. In recent years, laparoscopic approaches have been advocated [20].

## Conclusion

We report a case of a hepatic CSF pseudocyst secondary to the migration of a VP shunt, mistaken for hepatic hydatid disease with serious sequelae. The formation of a pseudocyst should always be considered in patients with VP shunts *in situ *and can be easily treated by simple repositioning.

## Abbreviations

CNS: central nervous system; CSF: cerebrospinal fluid; CT: computed tomography; IHA: indirect hemagglutinin; VP: ventriculo-peritoneal.

## Competing interests

The authors declare that they have no competing interests.

## Authors' contributions

WF drafted the manuscript; HHA and DM participated in the design of the study; MK participated in the design and coordination of the study. All authors read and approved the final manuscript.

## Consent

Written informed consent was obtained from the patient for publication of this manuscript and any accompanying images. A copy of the written consent is available for review by the Editor-in-Chief of this journal.
